# Oral healthcare provision at long-term care facilities in eThekwini: Perspectives of coordinators

**DOI:** 10.4102/phcfm.v15i1.3884

**Published:** 2023-06-20

**Authors:** Sonam Balwanth, Shenuka Singh

**Affiliations:** 1 Discipline of Dentistry, School of Health Sciences, University of KwaZulu-Natal, Durban, South Africa

**Keywords:** oral health provision, coordinator perspectives, long-term care facilities, caregivers, institutionalised residents

## Abstract

**Background:**

The prevalence and impact of oral disease among long-term institutionalised residents highlight the need for a scale-up of preventive and promotional oral health services that include oral health education and training for caregiving staff. However, opportunities to improve oral healthcare services are met with challenges.

**Aim:**

This study was undertaken to establish coordinator perspectives on oral health provision.

**Setting:**

Seven long-term care facilities in the eThekwini district, South Africa.

**Methods:**

An in-depth exploratory study was conducted with 14 purposively selected coordinators (managers and nurses). Semi-structured interviews were conducted and focused on coordinators’ experience and perspectives on oral healthcare. Data were analysed using thematic analysis.

**Results:**

The following themes emerged from the study: A lack of comprehensive oral health care practices, inadequate support from the dental sector, insufficient oral health prioritisation, limited funding for oral health, and challenges associated with coronavirus disease (COVID-19). All respondents reported that no oral health initiatives existed. Plans for oral health training workshops presented with coordination and funding challenges. Oral health screening initiatives have ceased since COVID-19.

**Conclusion:**

The study findings indicated that prioritisation of oral health services was inadequate. There is a need for continual oral health in-service training for caregivers and support from coordinators in guiding the implementation of oral health training programmes.

**Contribution:**

It is envisaged that the findings of this study will bring about greater coordinator involvement and collaboration with the public and private dental sectors to improve oral healthcare at long-term care facilities.

## Introduction

Long-term institutionalised residents constitute a vulnerable population with special oral health needs and therefore require greater attention and consideration when it comes to their oral health.^1^ One of the most important and basic needs is universal health coverage and access.^2^ While the World Health Organization (WHO) advocates for safe access to healthcare services for all sections of the population,^2^ especially vulnerable or marginalised groups, such as the elderly population, young children, and individuals with disabilities, this may not always be realised. Oral healthcare in South Africa is available through the private and public dental sectors.^3^ However, individuals residing at long-term care facilities may not always access dental services because of their reduced perceived need for oral care.^4^ Residents therefore rely on caregiving staff at long-term care facilities to see to their daily oral needs.^4^ The South African National Oral Health Strategy (2004) set out goals and targets intended at promoting and improving oral health for all citizens of the country.^5,6^ However, the policy overlooks important determinants of oral health, including the effect of comorbidities on individuals.^6^ In addition, the policy does not mention collaboration between healthcare professionals and both the private and public dental sectors, which could play a vital role in the prevention of oral disease among residents at long-term care facilities.^6,7,8^

Numerous international studies have indicated that oral health remains a neglected aspect of healthcare within long-term care facilities.^4,9,10,11,12^ The prevalence of oral disease among the institutionalised elderly is high.^13^ Common oral conditions experienced by the elderly include xerostomia, dental caries, periodontal disease and tooth loss, making it significantly difficult to masticate food, often resulting in nutritional deficiencies and leading to other systemic diseases.^10,13^ Similarly, institutionalised individuals with physical disabilities and learning difficulties were found to have periodontal disease.^11,14^ Furthermore, children residing in orphanages in India showed high rates of dental caries, periodontal disease and dental trauma.^15,16^ The presence of oral disease dramatically reduces an individual’s quality of life and negatively impacts their self-esteem.^13^ As a result of physical and mental limitations, long-term institutionalised residents may not be equipped to adequately perform oral hygiene for themselves and therefore depend on caregivers’ knowledge and skills to provide them with oral care.^10,11,12,17,18^

Caregiving staff play an essential role in maintaining oral hygiene and providing preventive and promotional oral healthcare to residents.^12^ However, numerous studies have reported a lack of adequate oral health-related knowledge and practices among caregivers to heed to the special oral health needs of the institutionalised residents.^9,11,12^ The lack of clear oral health guidelines and protocols within long-term care facilities creates a gap in the quality of oral healthcare services provided to institutionalised residents.^4^ International literature has identified important reasons for the high rate of oral disease and unmet oral health needs among institutionalised residents. Among these were: the disregard of the importance of oral hygiene among caregivers, time constraints to perform oral healthcare because of work demands and inadequate oral health knowledge among caregivers.^17,19,20,21^

There is a scarcity of research that unpacks the extent to which oral healthcare services are provided and the challenges and opportunities associated with the employment of oral health training workshops at long-term care facilities in the eThekwini district. Coordinator involvement in oral health planning, funding, logistics and support for oral healthcare initiatives is relatively unknown. Therefore, the purpose of this study was to gain a thorough understanding of coordinator perspectives on oral health provision at long-term care facilities in South Africa.

## Research methods and design

### Study design

This was an in-depth study which utilised an exploratory and qualitative study design, and formed part of a larger case study exploring the provision of oral healthcare at long-term care facilities. A case study design allows the researcher to explore the multifarious nature of complex phenomena at hand, and gain comprehensive understanding, by using multiple data sources and data collection methods.^22^

### Study setting

This study was conducted at seven long-term care facilities in the eThekwini district, KwaZulu-Natal, South Africa from March 2022 to August 2022. These long-term care facilities provide accommodation and meals for residents, in some facilities 24 h care, custodial care, healthcare services and basic oral healthcare services.^23,24,25^ The setting of this study was at six old age homes, which provide residential care and frail care to both the independent and dependent elderly population, chronically ill, those requiring palliative care, as well as the physically and cognitively impaired elderly population. The study was also conducted at one children’s home, which accommodates the orphaned, abandoned and vulnerable juvenile population from 2 years to 18 years of age. These long-term care facilities are located in the following areas within the eThekwini district: (1) Lamontville, (2) South Beach, (3) Berea, (4) Bluff and (5) Hillcrest. The majority of the participating long-term care facilities are non-profit organisations (NPOs), with the exception of one facility, which is privately funded. The operations of the long-term care facilities are primarily managed by the managers, nurses and caregivers.

### Study population and sampling strategy

A purposive sampling technique was utilised to recruit coordinators who were directly involved in the planning, implementation and clinical-coordination of oral healthcare services at each long-term care facility.^26^ Using a criterion and snowball sampling technique, the researcher identified potential participants who referred their colleagues to participate in the study.^26^ Those referred participants who met the inclusion criteria were selected. Coordinators who were not involved in the planning or implementation of oral healthcare services, who were not fluent in English, caregivers, funders, and volunteers were excluded in the sampling process. The study population consisted of four managers and 10 nurses, of which three nurses also performed managerial roles. Study sites were selected from ‘eThekwini health and well-being service provider directory 2018’ and a website called ‘Senior service retirement places’ on search engine company Google.^27,28^ Every facility was purposively included in the selection process.

### Data collection

Invitation letters were emailed to the coordinators of each selected long-term care facility, explaining the research study and requesting an appointment to conduct the interview. Twelve semi-structured interviews were conducted face-to-face and two interviews were conducted via Zoom meetings, because of the onset of coronavirus disease 2019 (COVID-19), and in keeping with strict COVID-19 protocols. Permission was obtained to audio record the process and the interview was conducted in English over a duration of approximately 30 min. Prior to conducting the Zoom interviews, an online link was emailed and guidelines were given to the two participants on how to use the application Zoom. All interviewees were requested to allocate approximately 1 h of quiet time, free from interruptions for the conduction of the interview. Participants were assured of their confidentiality, as each interview was conducted at a time and venue suitable to the interviewee. Each interviewee was anonymised and assigned an alphanumeric code to further assure confidentiality during the data analysis phase. The data were stored on the researcher’s personal laptop, which was only accessible to the researcher (S.B.) and supervisor (S.S.), thereby enhancing participants’ confidentiality.

A semi-structured interview guide was formulated by the researcher based on the research questions outlined in the larger case study and from a review of existing literature of similar studies.^29,30^ The interview guide was approved by Biomedical Research Ethics Committee of the University of KwaZulu-Natal. Open-ended questions were posed to participants, such as ‘What oral health initiatives exist at your long-term care facility’? and ‘Do you have any future oral health plans or interventions in the pipeline’? Participants were allowed to share their experiences on oral health initiatives offered to residents and caregivers at the long-term care facilities, oral health education and training opportunities for caregivers, support from the public and private dental sectors, existing oral health policy at long-term care facilities, the feasibility of implementing an oral health training workshop, and their perceptions on improving oral health services at long-term care facilities. The semi-structured interview approach permitted flexibility in the interview schedule, as open-ended questions allowed participants to provide meaningful explanations to the researcher and the researcher was able to ask follow-up questions.^31,32^ The researcher remained focused and was guided by the interview schedule, thereby prompting and encouraging the respondents to elaborate on their experiences and views.^33^ The number of respondents was not predetermined by the researcher, but data collection continued until data saturation was achieved.^33^ Previous studies suggested that data saturation is achieved after conducting 9 and 12 interviews, respectively.^34,35^ In order to reach meaningful saturation and a rich understanding of the subject matter, the researcher conducted 14 interviews.

### Data analysis

Interviews were audio-recorded, transcribed verbatim and observational and field notes were made by the researcher. The accuracy of the transcripts was confirmed by the researcher listening to the audio-recorded data again and re-reading the transcripts. Transcripts were typed into readable documents by the researcher, which were safely stored. An inductive coding approach was utilised, whereby the transcribed views and opinions expressed by participants were first cleaned and then analysed by a process called thematic analysis.^36,37^ This process included assigning codes to the data, identifying groups and sub-groups and ascertaining emerging themes, which were analysed using NVivo 12 software.^36,37^ As described by Braun and Clark, a methodical approach was utilised in organising and interpreting the qualitative data.^37^ Reading through the data allowed the researcher to become familiar with the participants’ perspectives.^37^ The first step involved the researcher sifting through the transcripts to retract relevant statements pertaining to the research topic and separating irrelevant statements. Applicable phrases and quotes of the transcripts were ‘colour highlighted’ to ‘correspond with codes’, which formed the second step. The initial coding frame was developed, which was then clarified, reviewed and sorted into groups.^37^ Code groups were then scrutinized for trends and emergent themes. The named themes were further examined to determine sub-themes (step three).^37^ Themes and sub-themes were cross-checked against the codes and transcript data by the supervisor, who has vast experience and expertise in qualitative research, thereby improving the credibility of the findings (step four).^38^ This contributed to the transferability of the data collected and allowed for extrapolation of the research findings to other similar settings.^38^ The authors clarified the finalised themes and sub-themes, and developed overarching themes reflecting the participants’ specific views and opinions on the research topic (step five).^37^

Different techniques were utilised to ensure the trustworthiness of the findings. Transferability was improved by providing a comprehensive description of the study methodology and dependability was ensured by keeping an audit trail.^38^ Member checking was performed with each participant to ensure that the collected data were a true reflection of the respondent’s views and opinions, which enhanced the credibility of the results.^38^ Checking and re-checking of the coding system by the researchers improved inter-coder reliability.^38,39^ The credibility of the findings was enhanced by the use of purposive sampling of participants and triangulation of the findings.^40,41^ The researcher is an oral healthcare professional involved in providing preventive and promotional oral healthcare services and education to patients of all ages and social backgrounds. Ensuring access to optimal oral healthcare for members of society, especially vulnerable individuals, is deemed a high priority for the researcher. The researcher’s personal values, beliefs and areas of potential bias were identified. Despite the researcher developing an amicable relationship with each of the respondents, no personal friendships were established. The researcher has no personal experiences with institutionalised residents, caregivers or coordinators at long-term care facilities. Reflexivity is ensured by limiting the researcher’s bias in conducting the research and the extent to which the researcher’s personal experiences, assumptions and beliefs influenced the research process and outcomes.^42,43^ The perceptions and views of the researcher remained neutral throughout the study, as the researcher detached from the opinions expressed by the participants. The researcher achieved this by practicing mindfulness and reflective thinking, by exploring similar research studies and expressing thoughts and opinions in a personal diary, and abiding by strict ethical principles.^44^

### Ethical considerations

Coordinators were informed that the study was voluntary and they were free to withdraw at any stage without any negative consequences. Written informed consent was obtained and the face-to-face and Zoom interviews were conducted with participating coordinators. Facilities and respondents have been anonymised to preserve confidentiality. The study was granted ethical clearance by the Biomedical Research Ethics Committee at the University of KwaZulu-Natal (BREC/00002633/2021).

## Results

The 14 coordinators (*n* = 14) (Table 1) were best suited to provide in-depth perceptions on the provision of oral health. This study defines coordinators as individuals, namely managers and nurses, who are directly involved in oral health policymaking and implementation of oral health activities at long-term care facilities. The four managers interviewed, provided pertinent information regarding oral health policy, managerial support, funding, logistics and feasibility of the implementation of oral healthcare programmes. The 10 nurses interviewed provided important information on the type of oral healthcare provided to residents, and were able to share their sentiments and that of the caregivers regarding oral hygiene knowledge and skills.

**TABLE 1:** List of coded respondents.

Long-term care facilities	Respondent Number	Role
Facility A	1	Nurse
Facility B	2	Nurse
Facility B	3	Manager
Facility C	4	Nurse
Facility C	5	Manager
Facility D	6	Manager
Facility D	7	Nurse
Facility E	8	Nurse
Facility F	9	Manager
Facility F	10	Nurse
Facility G	11	Nurse
Facility G	12	Nurse
Facility G	13	Nurse
Facility G	14	Nurse

The results indicated similar experiences and perceptions among respondents regarding oral healthcare at long-term care facilities. The themes emerging from the study included: A lack of comprehensive oral healthcare practices, inadequate support from the dental sector, insufficient oral health prioritisation, limited funding for oral health, and challenges associated with COVID-19.

### The lack of comprehensive oral healthcare practices

In response to the question: *What oral health initiatives exist at your long-term care facility?,* all interviewees reported that no oral health initiatives existed apart from caregivers providing the residents with basic oral health care services. All respondents indicated that the standard regime for oral healthcare included caregivers brushing the residents’ teeth twice a day in the morning and at night and performing mouth care by means of rinsing after meals. Some respondents indicated that basic denture care was also performed, which included rinsing the dentures after meals. However, flossing was not routinely practiced at long-term care facilities, as illustrated in the following comment:

‘We perform flossing only for those residents who have problems with their teeth.’ (Respondent 2, Facility B, Nurse)

Furthermore, mouthwash is seldom available and mainly used for residents under palliative care, where tooth brushing may not be possible. Respondent 8 highlighted the fact that there is insufficient oral health knowledge among caregivers regarding oral hygiene practices:

‘Flossing is not performed as much due to the cost of it and the lack of knowledge of flossing by the caregivers.’ (Respondent 8, Faculty E, Nurse)

All other respondents shared the same sentiments regarding caregivers’ lack of knowledge and skills in oral hygiene, flossing and denture care. Respondents acknowledged a gap in oral healthcare within their long-term care facility and attributed this to insufficient oral health education and training of caregivers.

### Inadequate support from the dental sector

The scarcity of oral health professionals from the public or private sectors visiting the facilities was a common finding among the interviewees. Respondents reported a need for support from oral health professionals in terms of conducting training workshops for the caregivers and performing preventive and promotional activities for the residents. In addition, respondent 6 reported that while dental sponsors sometimes visit the facility to deliver their products such as toothpaste or toothbrushes to the residents, no oral health activities were provided to the residents or caregivers. Similarly, respondent 1 commented:

‘We never had anyone come from the dental sector. If dental students come to perform oral screenings, it would be beneficial. Residents rely on their families to take them for their dental visits, as our facility does not have transport available. Many residents do not have medical aid and some family members are not present [gone overseas], therefore their oral health is neglected.’ (Respondent 1, Facility A, Nurse)

Respondent 8 expressed that while university students from other divisions of healthcare such as audiology and optometry visit residents and provide referrals for follow-up treatment, no dental students have come through to perform oral screenings for the residents. Likewise, respondent 6 commented:

‘Speech and hearing students come once a week to our home which has been incredibly helpful.’ (Respondent 6, Facility D, Manager)

Similarly, respondent 5 indicated that audiologists visit the facility twice a year and provide training to the caregivers on operating hearing aids, so as to provide efficient care to the residents. In addition, respondent 9 reported that an audiologist and optometrist come through to their facility to perform hearing and sight screenings for the residents and referrals to their private practice for follow-up treatment at a discounted rate.

Furthermore, respondents reported that on-site healthcare clinics do not offer comprehensive dental services and therefore simple oral ailments are managed by the nurses with varying levels of knowledge. Residents with more serious oral conditions are transported to the nearest government dental facility for follow-up treatment and those residents with medical aid are taken to a private dentist of their choice. Respondent 8 reported:

‘We are a NPO and none of the residents have medical aid, so if they do need to go to the government dental department, we do have transport available to take them.’ (Respondent 8, Facility E, Nurse)

A key point raised by respondent 6 was the challenge in accessing comprehensive dental services as dental treatment offered at government clinics are often limited to dental extractions. Respondent 2 further explained that dental services, which include dentures, are essential to help the elderly edentulous residents with chewing their food and restoring their self-esteem and confidence; however, this type of specialised dental services are not readily offered by the public health sector in eThekwini district.

### Insufficient oral health prioritisation

When asked the question: *Do you have any plans or oral health educational interventions in the pipeline?*, all respondents reported that participating in this current oral health research study was their first oral health educational intervention and indicated that no future plans for oral health education and training existed. When asked about implementing an oral health training workshop for caregivers, respondent 8 raised an important concern, stating that oral health is not perceived as an essential service by the caregivers and therefore the attendance may be low. This perspective is illustrated here:

‘The barrier would be getting the word out to get the caregivers to attend, as oral health is not perceived as being a big part of care for the elderly. Ideally, we would like more oral health education and promotional activities, but it is not prioritised at the moment.’ (Respondent 8, Facility E, Nurse)

According to respondent 1:

‘Participating in this research study was our first oral health initiative, outside of the regular in-service training.’ (Respondent 1, Facility A, Nurse)

Respondent 1 further reported that while oral health is on the agenda during in-service training, it is seldom conducted:

‘Oral health is on our schedule of topics during in-service training for the caregivers. We only do it once every 12 months or every 24 months.’ (Respondent 1, Facility A, Nurse)

Similar sentiments were expressed by respondent 13 who indicated that oral health is not specifically discussed unless caregivers receive complaints from residents regarding oral pain. Furthermore, in-service training is held approximately once every 2 months. This view is also reflected in the following statement:

‘If we do conduct training, it would be an in-service meeting of approximately 15 minutes. Oral health is not brought up and not prioritised at these meetings.’ (Respondent 13, Facility G, Nurse)

Respondents showed concern regarding the lack of oral health activities at their respective facilities and advocated for more awareness around oral healthcare and prioritisation within the long-term care facilities:

‘Yes I do think that there is always room for improvement. I think that oral care has been left out at long-term care facilities, as the residents seldom complain about dental problems. More awareness needs to be created around oral care within the long term care facilities.’ (Respondent 8, Facility E, Nurse)

Respondents indicated that insufficient attention has been given to oral healthcare at their facility and that oral screenings and training of caregiving staff would be beneficial in providing better care to the residents.

### Limited designated funding for oral health

The majority of the respondents indicated that their long-term care facilities are NPOs and thus receive a subsidy from the Department of Social Development of approximately R3000.00 per bed for sub-economic residents only. Other streams of income include residents’ South African Social Security Agency (SASSA) grant and donations. Funding is allocated towards 24 h care, board and lodging facilities including three meals a day and basic health care services. With regard to toiletries for oral care, respondent 9 reported:

‘The families would sometimes supply the products required or we as a facility would source the toothbrushes and toothpaste via donations. Economic residents provide their own toiletries for oral care.’ (Respondent 9, Facility F, Manager)

The only privately funded long-term care facility in the study does not receive donations or grants from the government, but instead receives funding directly from their residents, which caters for 24 h care, board and lodging services, healthcare and recreational services. With regard to the procurement of oral care toiletries at the privately funded long-term care facility, respondent 5 commented:

‘We rely on the family bringing toiletries like toothbrushes and toothpaste to the residents. If the family haven’t brought, then we keep stocks. We issue and charge the residents.’ (Respondent 5, Facility C, Manager)

All respondents reported that financing for oral health is not separate from the general funding for each facility and therefore oral health does not have a dedicated budget. When asked about the feasibility of implementing an oral health training workshop for caregivers, all respondents were optimistic and indicated that the space to conduct the training workshop was available, as well as co-ordination and support from management. This view was illustrated below:

‘We have the full support from management in co-ordinating the oral health programme with the dental professional.’ (Respondent 6, Facility D, Manager)

However, in terms of funding the workshop, respondent 11 responded:

‘There is no budget for oral health. Paying the oral health speaker would be a challenge.’ (Respondent 11, Facility G, Nurse)

Another respondent also reported that there is no specific oral health budget for training workshops, but travelling costs would be taken into consideration. Furthermore, a respondent added:

‘We rely on donations and sponsors. Government subsidies make up about 48% of the budget and used mainly for the upkeep of the building. Therefore, dental professionals coming in to perform the workshop will unfortunately not be compensated due to a lack of funds.’ (Respondent 6, Facility D, Manager)

Other respondents shared the same sentiments regarding the lack of funds to compensate oral health professional speakers and added that the budget for oral health training workshops is not prioritised.

All respondents reported that conducting oral health training workshops would be challenging because of time constraints and indicated that co-ordination of caregivers’ roster was necessary to accommodate other caregiving duties that take priority. Respondents reported that the caregivers’ roster is divided into two day shifts and two night shifts and therefore training workshops would need to be conducted in sessions to accommodate the caregivers’ duties so that care to the residents would not be compromised. A respondent further added:

‘In order to facilitate the oral health training, we would require the off duty caregivers to come on site, so it would impact on our budget as we would have to pay them for those hours.’ (Respondent 3, Facility B, Manager)

### Challenges associated with coronavirus disease 2019

One of the respondents reported that a road show was held in the area where oral screenings and referrals were conducted for the residents. However, since the emergence of the COVID-19 virus and implementation of restriction protocols, oral health programmes have ceased. Furthermore, another respondent noticed that caregivers’ attendance to an oral health training workshop could be affected because of the reduced capacity of gatherings. Respondents reported that despite the facility having a small budget for training and development, since the implementation of COVID-19 restrictions, training has been interrupted and put on hold.

## Discussion

The results of this study suggested an oversight of the importance of oral healthcare by coordinators and caregiving staff within long-term care facilities. This finding is supported by Petrovski et al. who reported that one of the reasons why oral health is neglected at long-term care facilities is related to the neglect in the acknowledgement of the importance and prioritisation of oral hygiene among nurses and caregivers.^4^ The reported inadequacy among caregivers in their oral health-related knowledge, attitudes and practices at long-term care settings indicated that there are significant gaps in coordinator awareness and involvement in addressing the special oral health needs of the institutionalised residents.

Of concern was the finding that standard oral healthcare services offered at long-term care facilities consisted mainly of brushing the teeth twice a day, rinsing the mouth of the edentulous residents and those under palliative care, and providing very limited denture care. The limited spectrum of oral healthcare services offered to the residents negatively impacts the residents’ oral health-related quality of life and in turn, overall well-being.^13,45^ Wener et al. found that poor oral health outcomes among institutionalised residents were found to be related to the fact that residents were solely dependent on their caregivers’ skills for daily oral care and have limited or elusive access to dental care as many rely on the long-term care facility to arrange access to external dental care.^46^ Similarly, the findings of this study indicated that caregivers lacked the knowledge and proficiency in performing adequate denture care and flossing for the residents. In addition, respondents stated that caregivers were only made aware of oral ailments experienced by the residents when extreme pain or discomfort was reported. Residents at long-term care facilities constitute a vulnerable population and thus require specialised oral healthcare services, including regular oral screenings and preventive activities to more comprehensive periodontal, restorative and prosthetic procedures.^4^ The results of this study indicated that oral health care is not prioritised or considered as an essential component of care, which would account for the lack of comprehensive oral hygiene care offered at long-term care facilities. Similarly, previous studies postulated that the general disregard for the provision of oral healthcare services at long-term care facilities was due: (1) to the heavy workload of caregivers (2) caregivers viewing the mouth as unpleasant, (3) considering oral care a cosmetic task, and (4) overestimating residents’ ability to independently perform their own oral care.^18,46,47,48,49^ Respondents also highlighted the fact that procuring toothbrushes, toothpaste and other additional oral aids were the responsibility of the resident and their families. Hence, the practice of flossing continues to remain a neglected aspect of oral hygiene care because of the cost implication on the resident and the lack of oral health awareness in terms of oral health instruction and education provided to the residents at long-term care facilities. Therefore, in order to achieve better oral health outcomes, the awareness of caregivers and coordinators regarding the increased oral health needs of the institutionalised becomes imperative.^4^ In this way, a clearer picture is created on performing appropriate oral hygiene practices and management of oral diseases and ailments.^4^

In this study, the absence of clear oral health guidelines for caregivers may be a contributing factor to a lack of prioritisation and motivation in providing optimal oral healthcare to the residents. In-service training provides a key opportunity to deliver oral hygiene instruction and education to the caregivers on a regular basis; however, the respondents have indicated that in-service training is rarely conducted at their facilities and oral health is often not on the agenda because of other pressing matters. Coordinator involvement, structured oral health protocols and targets would likely support and encourage caregivers to perform adequate oral care.

A study conducted by Stancic et al. in Serbia found that caregivers at long-term care facilities without oral care training, performed less oral hygiene procedures (brushing teeth, denture cleaning, rinsing with oral solutions) for the residents than those caregivers who had received oral care training.^10^ Therefore, oral health educational programmes and training are essential in improving the understanding of not only the caregiver but of the residents as well.^4^ The oral health-related knowledge, attitudes and behaviours that caregivers may acquire and develop following oral health training can have a significantly positive impact on the oral health of the residents.^50^ This finding is further confirmed by a study conducted by Wu et al. who found that an effective method in promoting the oral hygiene-related knowledge, skills and attitudes of the long-term care team was to reinforce oral health education.^18^ Similarly, Khanagar et al. found that there was a significant improvement in the oral health knowledge among the caregivers to the institutionalised elderly following an oral health educational programme.^51^ Therefore, upscaling oral health training and education among caregivers at long-term care facilities is necessary in ensuring better oral health outcomes for the residents.

Respondents indicated that there was a lack of dental support from the public and private sectors, as well as a paucity of preventive and promotional oral health activities offered to caregivers and residents. This finding suggests neglect of oral health awareness at long-term care facilities, as well as the lack of initiative and resourcefulness on the part of facility coordinators. The greater perceived need for hearing and sight screening raises a major concern and further emphasises the sidelining of oral healthcare within long-term care facilities. The responsibility of oral health promotion does not solely lie on the the health system, but also the organisation for promoting oral health within the facility.^52^ Oral healthcare contributes a vital component to the overall general well-being of individuals and thus oral screenings should be incorporated by caregivers as part of holistic care.^52,53,54^ Hecksher et al. found that adequate practical training in oral screenings among caregiving staff demonstrated that almost all participants were able to successfully recognise and manage oral diseases, as well as follow proper referral protocols.^55^ Prioritisation of oral examinations is thus important for prompt detection of oral disease, thereby reducing the advancement to complex oral diseases, which may in turn require more radical management.^54,56^ Integration of oral care to routine healthcare is imperative in providing effective preventive measures, management and referral for further dental care, thereby ensuring a better quality of life for the residents.^57,58^ There is a dire need to scale up coordinator involvement in promoting oral healthcare at long-term care facilities by collaborating with dental and medical professionals in both the public and private sectors.

The majority of the interviewees from the long-term care facilities reported to be NPOs, with the exception of one that was privately funded. An overriding theme was the general lack of funding, including funding for oral health activities and interventional programmes. With regard to residents, the majority cannot afford private dental care and oral care offered at the long-term care facilities are limited to management of minor oral ailments; thus, residents rely on the public healthcare system to see to their needs. There is a heavy dependency on the public oral health system, especially in developing countries such as South Africa and in underserved and marginalised populations.^59^ Primary oral healthcare facilities offer a basic package of dental care, which include: (1) dental examinations, (2) radiographs, (3) extractions, (4) simple restorative work, (5) scaling and polishing, and (6) emergency relief of pain.^5^ The private sector, on the other hand, offers a full range of dental services ranging from basic to specialised including dentures for the edentulous and preventive services for children and disabled individuals such as fissure sealants.^60^ For the public sector, providing comprehensive and tailored oral health services remains a low priority because of limited budgetary allocations, resulting in residents accepting less than ideal dental treatment.^61^ As the majority of the residents do not have access to private dental care, it often results in unmet oral health needs and in turn, a reduction in their quality of life.^13^ Therefore, the role of the nurses and caregivers is important in the early recognition of signs of oral disease so that they can make the necessary arrangements for appropriate preventive care.^4^

The lack of oral health preventive and promotional policies and activities within long-term care facilities is an oversight by coordinators and is a consequence of a lack of a dedicated directorate for oral health in the eThekwini district. This may be largely because of the lack of integration of oral health into policy and planning. Stronger collaboration is required between the health system^62^ and long-term care facilities for the provision of comprehensive oral healthcare service delivery. This may be achieved through outreach programmes collaborating with the private and public dental sectors, as well as the dental therapy school in University of KwaZulu-Natal (UKZN) to provide oral health preventive and promotional activities, oral screenings, management and referrals for the institutionalised residents. There is a need for advocacy initiatives to educate both coordinators and caregivers in terms of the importance of oral health through awareness campaigns on identifying specific oral diseases and conditions, management of the special oral needs of the residents, and the impact on overall well-being if the need is not addressed. The responsibility of providing oral healthcare at long-term care facilities should be a collective collaboration between health and oral healthcare workers, and all coordinators involved in general care provision.^62^ This will, in turn, enhance disease prevention and health promotion, thereby integrating oral health into overall general health.^62^

### Limitations of the study

The findings of this study provided insight into oral health provision at long-term care facilities in the eThekwini district. This study focused specifically on eliciting coordinator perspectives on oral healthcare services, and therefore its transferability may be limited to similar long-term care settings.^63^ Another limitation is that additional coordinators could have formed part of the study, such as coordinators from long-term disability rehabilitation centres. More research is required to determine the caregivers’ and residents’ perspectives on oral healthcare provision at long-term care facilities. Stakeholders such as policymakers, national regulators and civil society members should be considered in the inclusion criteria for future research on a larger and national scale.^63^

### Recommendations

The strategic planning of oral health policies, guidelines and protocols specific to the needs of institutionalised residents is non-prioritised on a national, provincial level and organisational level, implying that oral health is being disadvantaged in the budgetary and resources allocation.^64,65^ As a result, oral health education and training opportunities for caregivers and oral health preventive and promotional programmes for residents at long-term care facilities are overlooked, inevitably leading to poor oral health outcomes for residents. The improvement of oral health provision at long-term care facilities is unlikely to be effective without support and changes in the administrative and organisational culture.^30^ Recommendations can be made to national oral health planners to review oral health policies regarding marginalised populations residing within long-term care facilities to ensure that residents’ specific oral health needs are met. This will include advocating for mobile dental clinics that can provide preventive and promotional oral healthcare services, such as fissure sealants, fluoride treatments, and other routine dental procedures. On a micro level, long-term care facilities must re-align their oral health guidelines and protocols to include regular oral health in-service training and practical skills development for caregivers, so that caregivers are equipped to provide adequate preventive and promotional oral health activities to residents, including oral screenings and oral disease management. Coordinators of long-term care facilities can liaise with dental professionals and dental students to provide oral screenings to residents, and training to caregivers on performing oral examinations and managing oral diseases experienced by residents, as well as following proper referral protocols.^66^

## Conclusion

The results of the study indicated that oral health provision at long-term care facilities in the eThekwini district was neglected and non-prioritised. There is a need for continual oral health in-service training for caregivers, and a scale-up in preventive and promotional oral health activities within long-term care facilities, which can be achieved through guided implementation of oral health education and practical training for caregivers. Greater support, guidance and initiative from coordinators regarding oral health planning and policymaking, creation of oral health training opportunities for caregivers, and integration of oral health services to health policy is required to improve oral healthcare at long-term care facilities.
